# A Focus on Personalized Care in Light of New Diagnostic and Therapeutic Pathways of Shoulder and Elbow Disease

**DOI:** 10.3390/jcm14051425

**Published:** 2025-02-20

**Authors:** Teresa Paolucci, Alice Cichelli, Massimiliano Mangone

**Affiliations:** 1 Department of Oral Medical Sciences and Biotechnology, University of the Study G. D’Annunzio of Chieti and Pescara (UDA), Physical and Rehabilitation Medicine, 66100 Chieti, Italy; alice.cichelli@unich.it; 2 S. Spirito Hospital of Pescara, 65124 Pescara, Italy; 3 Center for Disability, Rehabilitation, and Sports Medicine, University of G. D’Annunzio of Chieti and Pescara, Physical and Rehabilitation Medicine, 66100 Chieti, Italy; 4 Department of Anatomy, Histology, Forensic Medicine and Orthopedics, Sapienza University, 00185 Rome, Italy; massimiliano.mangone@uniroma1.it

The shoulder is a complex system of functionally interconnected and coordinated joints. Together with the elbow joint and the hand, it guides and stabilizes the reaching movement of the upper limb. The functional role of the shoulder and elbow is twofold: to provide stability to the joint complex and to allow for a wide range of motion in the shoulder girdle and, through the elbow joint, to allow for quick and precise movements. These conditions—“stability”, “freedom of movement”, and “speed”—are lost if any of the numerous anatomical components are damaged by trauma, inflammation, or the peripheral or central nervous system.

Rotator cuff injuries, whether traumatic or due to functional overload, arthrosis, and heterotopic ossification, are the most common pathologies of the shoulder complex, while specific tendinopathies, with or without heterotopic ossification, are syndromes that specifically affect the elbow [1,2,3,4].

From these premises, the idea of promoting a Special Issue dedicated to “Diagnosis and Treatment of Shoulder and Elbow Disease” was born, which led to interesting reflections in light of the new diagnostic, surgical, and rehabilitative treatment proposals on this topic.

Eight papers were included in this Special Issue that address the topics presented in Figure 1.

There have been some insightful reflections on the use of Artificial Intelligence (AI) [5], in particular language models such as ChatGPT and Gemini, to support medical decision making in hand surgery. This underscores the importance of assessing the strengths and limitations of different models when incorporating them into clinical practice. However, more research is needed to evaluate the use and performance of additional AI tools (Pressman S.M. et al.). In addition, advances in arthroscopic techniques for repairing isolated subscapularis tendon tears [6] have become increasingly popular in recent years. Specifically, arthroscopic repair has been shown to achieve clinical and structural outcomes equivalent to the gold standard of open repair (Bartl C. et al.). Rehabilitation after rotator cuff repair is essential for functional recovery and to reduce the risk of retear [7,8]. The review by T. Paolucci et al. found that both early and delayed rehabilitation protocols after arthroscopic rotator cuff repair can provide adequate pain relief and functional recovery. However, early rehabilitation protocols generally result in better short-term improvements in range of motion and strength. These benefits may not last, and the risk of recurrence remains a concern with early rehabilitation, especially for major injuries. Clinicians should carefully evaluate each patient’s individual characteristics, injury severity, and specific therapy modalities to determine the most appropriate rehabilitation protocol following rotator cuff repair. Shoulder pain is a common musculoskeletal condition, accounting for approximately 1.3% of all primary care visits. When conservative and pharmacologic treatments fail, arthrodesis is typically considered a salvage procedure with limited functional goals. However, in “selected” patients, it can provide effective pain relief, stable motion, and satisfactory functional outcomes [9]. In the patient series by Sobhi, S. et al., shoulder arthrodesis demonstrated remarkable efficacy in pain reduction, high patient satisfaction, and acceptable functional outcomes with manageable complication rates. Considering that chronic shoulder pain can lead to significant functional disability and reduced psychosocial well-being, often exacerbated by kinesiophobia (Alito A. et al.), these findings highlight the potential benefits of this procedure in appropriately selected patients. Pathology of the long head of the biceps (LHBs) tendon is a common cause of anterior shoulder pain, including tendinitis, tenosynovitis, subluxation, dislocation, and tears [8]. In the pathogenesis of LHBs tendon instability, a shallow intertubercular groove morphology has been identified as a potential predisposing factor. Gerhardinger K. et al. suggested that the dimensions of the LHBs tendon may also influence instability, and they introduced the tendon-to-groove cross-sectional area ratio as a novel parameter to describe individual local anatomy. Heterotopic ossification (HO) after elbow trauma can result in significant motion restriction, depending on the size and location of the HO, as seen on radiographs [10]. On this specific topic, Leyder D. et al. introduced a novel classification that provides more detailed insights compared to previously established systems and aims to assist clinicians in making treatment decisions. Finally, Longo U.G. et al. proposed a minimal clinically important difference (MCID) for the 36-item Short-Form Health Survey (SF-36) questionnaire to assess patient-acceptable symptom status (PASS) during both preoperative evaluation and final follow-up after surgical shoulder repair.

We hope that you enjoy reading the articles published within this Special Issue. 

## Figures and Tables

**Figure 1:**
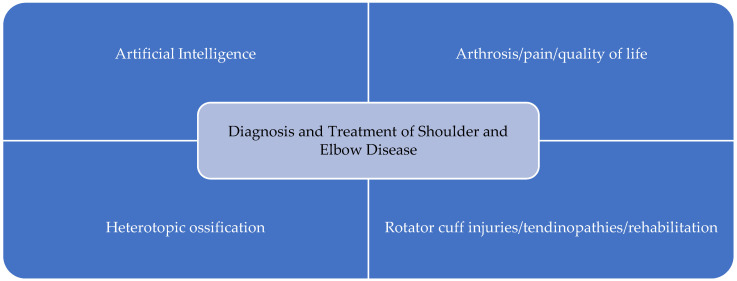
Topics of the included papers.

## References

[1] Mall N.A., Chahal J., Heard W.M., Bach B.R., Bush-Joseph C.A., Romeo A.A., Verma N.N. (2012). Outcomes of arthroscopic and open surgical repair of isolated subscapularis tendon tears. Arthroscopy.

[2] de Sire A., Agostini F., Bernetti A., Mangone M., Ruggiero M., Dinatale S., Chiappetta A., Paoloni M., Ammendolia A., Paolucci T. (2022). Non-Surgical and Rehabilitative Interventions in Patients with Frozen Shoulder: Umbrella Review of Systematic Reviews. J. Pain Res..

[3] Li L., Sun Y., Qin H., Zhou J., Yang X., Li A., Zhang J., Zhang Y. (2023). A scientometric analysis and visualization of kinesiophobia research from 2002 to 2022: A review. Medicine.

[4] Heuts P.H., Vlaeyen J.W., Roelofs J., de Bie R.A., Aretz K., van Weel C., van Schayck O.C. (2004). Pain-related fear and daily functioning in patients with osteoarthritis. Pain.

[5] Miller R., Farnebo S., Horwitz M.D. (2023). Insights and trends review: Artificial intelligence in hand surgery. J. Hand Surg. Eur. Vol..

[6] Nazari G., MacDermid J.C., Bryant D., Dewan N., Athwal G.S. (2019). Effects of arthroscopic vs. mini-open rotator cuff repair on function, pain & range of motion. A systematic review and meta-analysis. PLoS ONE.

[7] Littlewood C., Bateman M., Butler-Walley S., Bathers S., Bromley K., Lewis M., Funk L., Denton J., Moffatt M., Winstanley R. (2021). Rehabilitation following rotator cuff repair: A multi-centre pilot & feasibility randomised controlled trial (RaCeR). Clin. Rehabil..

[8] Van Deurzen D.F.P., Garssen F.L., Kerkhoffs G.M.M.J., Bleys R.L.A.W., Ten Have I., van den Bekerom M.P.J. (2021). Clinical relevance of the anatomy of the long head bicipital groove, an evidence-based review. Clin. Anat..

[9] Del Core M.A., Cutler H.S., Schacherer T., Khazzam M. (2021). Glenohumeral arthrodesis. JSES Rev. Rep. Tech..

[10] Wiggers J.K., Helmerhorst G.T., Brouwer K.M., Niekel M.C., Nunez F., Ring D. (2014). Injury complexity factors predict heterotopic ossification restricting motion after elbow trauma. Clin. Orthop. Relat. Res..

